# Can Rational Emotive Behaviour Therapy (REBT) and Mindfulness be Integrated Effectively within High Performance Settings?

**DOI:** 10.1007/s10942-022-00475-x

**Published:** 2022-08-23

**Authors:** Paul Young, Vivien Chow, Cheryl Haslam, Andrew Wood, Jamie Barker

**Affiliations:** 1 School of Sport, Exercise and Health Sciences, Loughborough University, Loughborough, UK; 2 School of Architecture, Building and Civil Engineering, Loughborough University, Loughborough, UK; 3 Faculty of Health, Psychology and Social Care, Manchester Metropolitan University, Manchester, UK

**Keywords:** REBT, Mindfulness, Performance, Psychology

## Abstract

Our critical commentary explores the overlaps and divergences between Rational Emotive Behaviour Therapy (REBT) and contemporary mindfulness practice and considers whether the approaches could be integrated and applied effectively within two high performance settings in particular: sport and business. It highlights how REBT and mindfulness share similar philosophical positions on the causes of emotional disturbance, on the importance of acceptance, and on cultivating self-awareness to respond healthily and adaptively to adverse events. It also acknowledges diverenges relating to REBT and mindfulness’ respective positions on judgement of thoughts, an emphasis on the present moment, and meditative practice. We observe that by cultivating metacognitive awareness—a capacity to impartially observe thinking—mindfulness may help individuals in high performance settings to see more clearly how their beliefs influence their emotional, cognitive, and behavioural outcomes. Mindfulness could thus potentially aid an individual’s transition from intellectual insight to emotional rational insight within an REBT framework. Moreover, appropriately integrated mindfulness practice alongside REBT-based work may help individuals within high performance settings, and beyond, to cultivate a mindset that is grounded in the present, less distracted and more task focused, potentially enhancing performance outcomes.

## Introduction

### REBT and Mindfulness: A Brief History

Rational Emotive Behaviour Therapy (REBT) is a cognitive behavioural approach to psychotherapy developed by the American psychologist Albert Ellis (Ellis, 1957, 1962). Ellis created Rational Therapy (RT)—later renamed Rational Emotive Therapy (RET), then, from 1995, REBT—having become disillusioned with psychoanalytic methods in the Freudian tradition which he considered inefficient and untargeted (Ellis, 1995; Still & Dryden, 1998). Using the acronym REBT for all iterations of Ellis’ therapeutic approach, REBT was the first model of psychotherapy to describe how emotional disturbances result from dysfunctional thinking in response to adversity, and to specifically target the disputation and replacement of irrational beliefs with rational beliefs as a primary mechanism for positive emotional change (David et al., 2005). REBT is considered by many to be the original form of Cognitive Behavioural Therapy (CBT), having been introduced chronologically prior to the preeminent Aaron T. Beck’s early work on cognitive factors in depression which was the precursor to his Cognitive Therapy (CT; Beck, 1963, 1964). Together, REBT and CT were the two pillars underpinning the ‘second wave’ of CBT whereby cognitive and behavioural approaches to psychological treatment became integrated (Thoma et al., 2015).

REBT and CT share several fundamental similarities and, Ellis suggested, have become more similar over time (Ellis, 2003b). Crucially, both REBT and CT consider thinking, feeling, and behaving as “integrally and interactionally related” (Ellis, 2003b, p. 227). For the purposes of our commentary, particularly when discussions turn to the ‘third wave’ of CBT, it is important to note that REBT and CT are also distinctive approaches with key differences. A detailed assessment of these differences is beyond the scope of this commentary. However, in general terms, REBT is philosophical, humanistic, and largely transdiagnostic whereas CT is rooted in empiricism, scientific reality testing and is more closely tied to psychiatric diagnoses (Ellis, 2003b). REBT focuses on disputing deeply held irrational beliefs which include primary ‘must’ demands, and secondary beliefs relating to awfulizing, low frustration tolerance and self, other and world damning (Ellis & Dryden, 2007). REBT eschews disputing or reality testing higher order thinking processes, such as inferences and negative automatic thoughts, because Ellis considered replacing irrational beliefs with rational beliefs to be most crucial for lasting emotional, behavioural, and psychological change (Ellis & Dryden, 2007). In contrast, CT is primarily concerned with correcting faulty higher order thinking and the functionality of a client’s beliefs within their own value systems and life experiences rather than directly assessing their rationality against pre-ordained ideas (Padesky & Beck, 2003).

Although CT incorporates core belief, or schema, work within its framework, it typically addresses higher order thinking over or before such work. Crucially, CT does not actively incorporate philosophy into its therapeutic process in the way that REBT does (Ellis, 2005). What has become widely known as ‘traditional CBT’ arguably reflects Beck’s CT with integrated behavioural treatment aspects more so than it does Ellis’ REBT, for several reasons not least that it excludes REBT’s philosophic emphasis. As Ellis (2005) put it, “traditional CBT is far from being the same as REBT” (p. 161). The third wave of CBT, which has risen to prominence in the past two decades, has seen the integration of mindfulness meditation and related practices with aspects of CT (as opposed to REBT) for the treatment of an array of psychological disorders including depression and anxiety (e.g., Segal et al., 2002).

Western interest in mindfulness philosophy, and mindfulness-based meditation specifically, has grown rapidly in recent decades (Baminiwatta & Solangaarachchi, 2021). Secular mindfulness practice—distinct from mindfulness in the Buddhist tradition but retaining many of its components—has become incorporated into numerous Western psychological therapies (Öst, 2008), and popular apps offering guided mindfulness meditation continue to attract millions of subscribers (Perez, 2020). Mindfulness features prominently in many ancient Buddhist traditions including Theravada, Mahayana, and Vajrayana, but the roots of meditative mindfulness practice found in contemporary Western settings can largely be traced back to Tibetan Buddhism (Dredze, 2020). In Buddhist teachings, mindfulness is seen as one of the ways in which to cultivate self-knowledge and wisdom required to reach the goal of enlightenment, whereupon freedom from suffering is the ‘reward’ for letting go of unhealthy desires and attachments (Keng et al., 2011).

Mindfulness is a multifaceted concept, and some have observed that this can give rise to confusion regarding what it encompasses (Baer, 2003; Roemer et al., 2003). The word ‘mindfulness’, for example, is said to simultaneously represent a specific psychological trait, a mode or state of awareness and the practice of cultivating mindfulness itself through meditation (Siegel et al., 2009). While the umbrella definitions of mindfulness cover a range of related sub-concepts, present moment awareness and non-judgemental acceptance are recognised universally as being central features of mindfulness, and meditation is the most common but not the only method of contemporary mindfulness practice (Baer, 2003; Kabat-Zinn, 1990).

Some scholars, including Albert Ellis, have noted similarities between the teachings of mindfulness and REBT, going so far as to suggest that they may have integrative potential (e.g., Ellis, 2005, 2006; Whitfield, 2006). This is perhaps not surprising, given the integrative potential of mindfulness and CT, which is arguably less philosophic than REBT, has already been indicated within third wave CBT approaches (e.g., Segal et al., 2002). However, some authors have cautioned against the integration of mindfulness and CBT, of which REBT is one modality, on the grounds that mindfulness and the core principles of CBT are incompatible because the former takes a non-judgemental approach to thoughts while the latter includes cognitive restructuring, and that the concept of mindfulness outside of its Buddhist religious tradition lacks meaning (e.g., Harrington & Pickles, 2009). It is an interesting debate which acts as a backdrop to our critical commentary.

### Aims of this Commentary

The main aims are threefold. First, to highlight the theoretical and philosophical overlaps, as well as divergences, between REBT and mindfulness. Second, to explore the theoretical and applied appropriateness of integrating mindfulness practice within REBT protocols broadly. Third, to consider whether high performance settings, specifically the realms of sport and business, could be appropriate contexts in which to deliver mindfulness integrated REBT interventions. Beforehand, we shall consider further the history of REBT and mindfulness to outline how both have separately come to be considered as potentially effective interventions in high performance settings, having started out in clinical contexts.

### From Clinical to High Performance Settings

REBT and contemporary applications of mindfulness have followed broadly similar trajectories since their introduction to Western psychotherapy in the mid and late twentieth century respectively having both initially been conceived as clinically oriented psychotherapeutic interventions (Ellis, 1957, 1962; Kabat-Zinn, 1982) before extending into non-clinical and performance-related areas. The development of REBT, and to a large degree its success, has been driven by its founder Albert Ellis, for whom delivering, writing about, and teaching REBT was a life’s work. But if there is perhaps one area of weakness for REBT, as Ellis himself acknowledged, it is an evidence base that although not unconvincing, is not as deep as it might be given its longevity, and is certainly thinner than that of Beck’s CT (Neenan, 2001). Nevertheless, a recent meta-analysis of 84 REBT research studies in clinical, sub-clinical and non-clinical settings, conducted over the last 50 years, concluded that that REBT was “a sound psychological intervention” based on a medium overall effect size (*d* = 0.58) for REBT compared to other interventions across outcomes at post-test (David et al., 2018, p. 304).

The adoption of mindfulness-based interventions within secular Western clinical settings began with the work of Jon Kabat-Zinn who initially developed a Mindfulness-Based Stress Reduction (MBSR) programme for patients living with chronic pain (Kabat-Zinn, 1982). Kabat-Zinn’s work was the catalyst for the integration of mindfulness with elements of CT for the subsequent treatment of depression, anxiety, and borderline personality disorder, among other disorders, often referred to collectively as the third wave of CBT. Notable third wave CBT approaches include Mindfulness Based Cognitive Therapy (MBCT; Segal et al., 2002), Acceptance and Commitment Therapy (ACT; Hayes et al., 1999) and Dialectical Behaviour Therapy (DBT; Linehan, 1993). Since their introduction in the 1990’s, third wave approaches have become influential within the CBT movement and have been adopted alongside traditional CBT approaches within health systems such as the UK’s National Health Service (NHS; Tickell et al., 2020). Findings from meta-analyses of randomised controlled trials (RCTs) generally support the efficacy of third wave CBT approaches across a range of clinical settings and disorder types (e.g., Kuyken et al., 2016), although the methodological robustness of some RCTs have been questioned (see Öst, 2014).

In recent decades, researchers have come to consider that both REBT and mindfulness may have potential to enhance psychological performance within high performance settings. From the perspective of REBT, this potential is seen to reside in the benefit to high performing individuals of holding rational beliefs over irrational beliefs. From the perspective of mindfulness, cultivating a mindset of non-judgemental awareness of the present moment is hypothesised as being beneficial. Most REBT research within high performance settings has been undertaken within the context of sport, although even here the literature is in its infancy (see Turner, 2019). Encouraging outcomes from REBT have been observed across a range of sporting contexts including soccer (Turner et al., 2014), Paralympic soccer (Wood et al., 2018), archery (Wood et al., 2017), cricket (Turner & Barker, 2013) and squash (Deen et al., 2017). Beyond sport, there is a smaller but growing literature on REBT’s application in other high performance settings including business (e.g., Turner & Barker, 2015), emergency services personnel (Jones et al., 2021; Wood et al., 2021) and the military (Jarrett, 2013), but it is too early for firm conclusions regarding its efficacy in these areas.

The notion that mindfulness practice may enhance mental performance and improve goal-directed outcomes has been investigated in many of the same high-performance settings as REBT, namely sport (e.g., Bühlmayer et al., 2017), business (e.g., Bostock et al., 2019) and the military (e.g., Brewer, 2014). The early signs have been encouraging, particularly in sport and business where most of the research has been conducted (Bühlmayer et al., 2017; Vonderlin et al., 2020) but, as with REBT and performance, the literature remains in its formative years. A meta-analytic review of nine trials noted that mindfulness practice was linked with decreased psychological stress within sporting settings which the authors noted could lead athletes to more goal-oriented performance (Bühlmayer et al., 2017). Similarly, a larger meta-analysis of 56 studies conducted by Vonderlin and colleagues (2020) identified that mindfulness-based programmes were effective in promoting the health and wellbeing of employees in various occupational settings including, but not limited to, business. Interest in mindfulness applied to high performance settings is growing rapidly, but there is a widely accepted need for more methodologically robust work—such as randomised controlled studies with active control groups—before firmer conclusions on efficacy can be drawn (Bühlmayer et al., 2017).

In sum, REBT and mindfulness-based approaches have evolved similarly in that both are now being applied within high performance settings having been originally developed for the clinical treatment of psychological disorders. To date however, despite rare exceptions (e.g., Chenneville & St John Walsh, 2016; Chenneville et al., 2017), there is a paucity of literature on ‘pure’ REBT (to distinguish between Ellis’ REBT and Beck’s CT) and mindfulness having been actively integrated within clinical or indeed high-performance settings.

## Overlaps Between REBT and Mindfulness

Scant evidence of combining REBT and mindfulness in the literature is interesting, given that CT has been integrated successfully with mindfulness in third wave CBT approaches (Segal et al., 2002), and the philosophical overlaps between REBT and mindfulness are, if anything, more overt than with CT (Hayes, 2005). These overlaps have been noted by numerous scholars, including Albert Ellis himself (see Ellis, 2005, 2006; Holt & Austad, 2013; Whitfield, 2006). Ellis had suggested that REBT and ACT—which employs mindfulness techniques within its treatment framework and is influenced by Buddhist philosophy—could potentially integrate (Ellis, 2005). In one of his last published papers, Ellis noted that REBT and MBSR had much in common, particularly in relation to the importance of acceptance in bringing about therapeutic change (Ellis, 2006). Others have also observed similarities between REBT and ACT (e.g., Ciarrochi & Robb, 2005; Ciarrochi et al., 2005). Indeed, the founder of ACT Stephen Hayes noted that “of all the CBT approaches, REBT seems most compatible with the primary thrust of third wave interventions” (Hayes, 2005, p. 140), but Hayes also considered irrational belief disputation to be incompatible with ACT’s core principle that context and response, not the content of thought is most important. Thus, integration in a formal sense was never achieved.

In the following sections we will examine the apparent overlaps between REBT and mindfulness, as well addressing areas of divergence. Overlaps, or similarities, have been identified broadly as relating to their respective philosophical positions on the causes of disturbance, themes of acceptance, and a similar focus on cultivating awareness to respond to events in a rational, adaptive manner. Divergences identified relate to positions on the need for judgement of thinking, the present moment, and the need for meditation. Later, informed by this exploration, we postulate the ways in which REBT and mindfulness might be used together within high performance settings to enhance psychological performance and wellbeing.

### Philosophic Positions on the Root Cause of Emotional Disturbance

REBT and mindfulness share similar overarching philosophic positions on the cause of lasting emotional disturbance: responses to events, not events themselves. Ellis was famously inspired by the works of ancient philosophers, particularly Roman and Greek stoics such as Marcus Aurelius and Epictetus, who was recorded in *Enchiridion* (translated from Latin: ‘Handbook’) as saying: “Men are disturbed not by things, but by the views which they take of them” (Long, 1991). Ellis aligned REBT closely with this dictum, offering an ABC model to demonstrate how disturbance at ‘C’ results not from adversity at ‘A’, but rather the views, or beliefs (at ‘B’), held about the adversity (Dryden, 2005). REBT points to irrational beliefs (at ‘B’) as underpinning emotional disturbance and argues that disputing and replacing irrational beliefs with rational beliefs leads to healthier and more adaptive emotional, behavioural, and cognitive outcomes. For individuals to achieve emotional change with REBT however, they must first acknowledge its general principle of emotional responsibility which reflects Epictetus’ dictum and is captured by Dryden (1995): “We are largely, but not exclusively, responsible for the way we feel and act by the views we take of the events of our lives” (p. 11).

There are notable alignments between REBT’s position on how individuals disturb themselves (i.e., from views of events, not events themselves) and that of contemporary mindfulness which derives its philosophical underpinnings from Buddhism. This alignment is notable within the Buddha’s famous ‘two arrows’ parable (see Teasdale & Chaskalson, 2011) in which the Buddha described how lasting unhappiness and suffering derives not from the first metaphorical ‘arrow’ of unwelcome adverse events but rather from the second ‘arrow’ that we strike unto ourselves in how we *respond* to the thoughts and feelings stirred up by such events. Contemporary Western adaptations of mindfulness encapsulate the Buddha’s message, teaching that individuals have agency over their emotional reactivity which resides in a capacity to choose how to respond to mental and physical events. There are evident parallels which can be drawn between this philosophy and that of REBT.

### Awareness of Reality versus Illusion

A further commonality between REBT and mindfulness is that both approaches aim to help individuals see things ‘clearly’, so that they can respond to events in life in a manner which is healthy and proportional with reality. REBT emphasises the importance of rational thinking; of holding flexible preferences over rigid demands and assessing adversity—and one’s capacity to cope with discomfort arising from it—from a logical, realistic standpoint (Ellis & Dryden, 2007). REBT encourages clients to become aware, through contemplative session-based and individual homework, of the direct relationship between their beliefs at ‘B’ and the emotional, behavioural, and cognitive outcomes they experience at ‘C'. Successful REBT clients may become, in effect, masters of their own belief systems (Ellis & Dryden, 2007).

This is easier said than done, however, for Ellis considered humans to be pre-disposed to irrationality, making challenging and correcting irrational thinking and behaviour difficult:

Humans have a strong tendency to needlessly and severely disturb themselves, and that, to make matters much worse, they also are powerfully predisposed to unconsciously and habitually prolong their mental dysfunctioning and to fight like hell against giving it up (Ellis, 1987, p. 365)

In keeping with REBT’s perspective on human pre-disposition to irrationality, mindfulness scholars describe the human mind as having an ‘illusionary’ tendency with which we all continuously contend (Wright, 2017). By this it is meant, in simple terms, that the way in which we perceive ourselves (including our inner thoughts, emotions, and sensations), others and the world around us is not always (in fact often not) entirely aligned with reality. A possible reason for the misalignment is that the evolutionary goal of humans to pass on their genes above all else, remnant of the hunter gatherer societies of our genetic forebearers, bears little resemblance to the rational thought, feeling, and behaviour relevant to the twenty-first century society (Wright, 2017). Mindfulness practice, it is said, helps individuals to overcome these ‘illusionary tendencies’ by teaching, and experiencing through training in awareness, that thoughts and feelings are (a) not facts, (b) transitory and impermanent, and (c) not defining of the self (Mellinger, 2010). Capturing part of the essence of mindfulness, and perhaps unwittingly revealing an overlap between it and REBT in the process, Wright (2017) wrote: “Mindfulness meditation is, among other things, a tool for examining our stories carefully, from the ground up, so that we can, if we choose, separate fact from fiction” (p. 152).

A term used by mindfulness theorists to describe a capacity to perceive and be aware of one’s own thoughts from an observer standpoint is ‘metacognitive awareness’ (Teasdale et al., 2002, p. 275). It has been suggested that both mindfulness and CBT, of which REBT is a foundational modality, share common ground as metacognitive strategies, because they both “consist of intentional and automatic efforts that individuals devote to controlling their cognitive activities and for distinguishing and separating rationality from irrationality” (Mellinger, 2010, p. 234). In line with this position, Whitfield (2006) described mindfulness as being “a concept that can be applied to enhance the patient’s perceptions and understanding of the realm of the B [beliefs], a world that the patient can increase his control of” (p. 208). He noted that mindfulness could potentially help individuals working within an REBT treatment framework to see, through practice, that “mental and bodily events rise from ourselves, and can diminish irrespective of what is going on in the outside world” (p. 209). The notion, as posited by Whitfield (2006), that mindfulness training could help individuals to become more aware of their beliefs at ‘B’ and to experientially witness the resultant differences in emotional outcomes at ‘C’, dependent upon their rationality, appears to offer at least one potential theoretical route to the integration of REBT and mindfulness.

### Acceptance

Many scholars have observed that REBT and mindfulness are most aligned in their mutual emphasis on acceptance (e.g., Whitfield, 2006). Ellis himself noted the commonality: “REBT and Kabat-Zinn’s MBSR both heavily favour unconditional self-acceptance (USA) combined with unconditional other acceptance (UOA) and unconditional life acceptance (ULA)”, he wrote when comparing REBT with MBSR (Ellis, 2006, p. 77). Ellis was forthright in his criticism of the concept of self-esteem which he considered the antithesis of acceptance, calling it the “the greatest sickness known to man or woman because it is conditional” (Epstein, 2001, p.71). He argued that to attempt to rate, or ‘esteem’, the ‘self’ was illogical because humans are infinitely multi-faceted and that there is no one overall entity to rate. Individuals, he argued, should instead ‘‘fully accept themselves as valuable and enjoyable human beings whether or not they are self-efficacious and whether or not others approve of or love them’’ (Ellis, 1996, p. 150). Ellis also noted overlap between REBT’s perspective on the irrationality of self-rating and Buddhist perspectives on the dissolution of the ego and a fixed sense of self (Thompson & Waltz, 2008). Buddhists, Ellis was aware, also consider the idea that there is a permanent, unchanging self to be an illusion because nothing remains constant, everything is always changing; a position with notable similarities to REBT’s rational, logical stance on acceptance.

It seems reasonable to postulate that mindfulness practice may help to strengthen USA, UOA and ULA among REBT clients when working on irrational beliefs relating to acceptance across a variety of settings. Others have also made this observation. For example, having conducted a study among 167 university students which indicated that everyday mindfulness is related to unconditional self-acceptance, Thompson and Waltz (2008) noted that “mindfulness skills may offer a means to cultivate unconditional self-acceptance and to shift from an emphasis on self-esteem as a measure of worth” (p. 119). Within the context of unconditional acceptance in the REBT framework, mindfulness practice may support REBT by allowing clients to observe and accept the reality of the presence of unwanted cognitions and feelings as they arise at ‘C’ in the ABC model. Accepting thoughts and feelings at ‘C’ could potentially reduce maladaptive rumination, allow for more adaptive behaviour in the present moment, and provide additional mental capacity and malleability for appropriately timed cognitive belief restructuring work.

There may be linkages between this idea and the Buddhist concept of ‘letting go’, which is captured within the ACT model and is sometimes referred to as turning off the “struggle switch” (Harris, 2006, p. 6). A skilful practitioner using REBT and mindfulness in combination within performance settings may be able to apply timely active and focused irrational belief disputation work alongside teaching clients to mindfully observe and allow, rather than ‘struggle’ against, unwanted thoughts and feelings in the present moment. By way of an example, an elite athlete could be encouraged to challenge the validity and functionality of their rigid unhealthy beliefs about having to win at all costs, whilst simultaneously adopting an unconditionally accepting or allowing attitude towards unwanted feelings and automatic thoughts they experience in the present moment, say as they take to the starting blocks in a key race.

## Divergences Between REBT and Mindfulness

In the following sections we consider the potential divergences between REBT and mindfulness, as identified within the extant literature, and scholarly attempts to bridge these theoretical gaps. The divergences discussed relate to positions on judgement of thoughts, an emphasis on the present moment, and meditative practice.

### Judgement versus Non-Judgement

REBT and mindfulness deviate somewhat with regards to the notion of whether judgement, in particular judgement of thoughts, is necessary to enhance emotional and psychological health. Mindfulness philosophy holds that thoughts (and feelings) should be observed without any attempt to evaluate, alter, or change their content and caution that disturbance in fact often *follows* attempts to avoid, suppress or over-engage with distressing thoughts, emotions, and feelings (Hayes & Feldman, 2004; Kabat-Zinn, 1990). Mindfulness has thus been described as a radical form of acceptance (Linehan, 1993), moving beyond tolerance towards active embracing of present-moment experience, absent of judgement altogether (Hayes, 2004). Contrastingly, Ellis argued that we should evaluate our beliefs to identify and replace those that are irrational with rational alternatives. The process of reviewing, disputing and actively seeking to change thinking processes within a cognitive behavioural model is called cognitive restructuring (Ellis, 2003a).

At first glance, REBT’s position on cognitive restructuring may seem unresolvable with that of mindfulness which promotes non-judgement. However, Ellis noted that through mindful observation, clients can come to realise that much of their thinking is irrational and unhelpful, offering a bridge between the two perspectives (Ellis, 2006). Ellis noted, “MBSR looks for – as does REBT – mental (and emotional) distortions, examines them, sees how confusing they are, and seeks out methods to minimize them in real-life, practical solutions” (Ellis, 2006, p. 66). Furthermore, Mellinger (2010) noted that both CBT and mindfulness “distinguish adaptive from maladaptive metacognitions on the basis of their accuracy in perceiving the external and internal environment, and both emphasise the importance of correcting erroneous perceptions and conceptions” (p. 234). Hence, mindfulness practice may elicit belief change in a similar fashion to REBT, but through an implicit rather than explicit process. In a paper revisiting Ellis’ writing on REBT and MBSR, Dredze (2020) observed that even without including disputation, Ellis appreciated mindfulness as a potential means of recognising irrational thoughts, an important first step in the thought restructuring process. Indeed, by encouraging participants to see thoughts not as facts, mindfulness may help to loosen the grip of irrational beliefs, which can to the holder seem very much like fixed truths. An example of this in a performance context such as business could be when an executive is encouraged to step back and examine with curiosity—in the knowledge that thoughts are not facts—their recurring upsetting thought that they should be more successful than they are.

### The Present Moment

A further apparent area of divergence between REBT and mindfulness relates to their perspectives on the present moment. As with acceptance, mindfulness takes the more extreme position. Mindfulness practitioners do not dismiss future and past-oriented thinking as an essential part of human experience but warn that individuals become disturbed when they get stuck in past or future based ruminative patterns of thinking. Mindfulness-based therapies thus encourage individuals to bring awareness, through meditative practice, to the current moment specifically and purposely to train the mind’s capacity to notice when it has become preoccupied in daily life (Kabat-Zinn, 2009). Similarly, REBT targets the mechanisms that maintain emotional disturbance in the present (namely, beliefs at ‘B’ in the ABC model) and, without dismissing its influence, typically shies away from focusing on past childhood and/or developmental events as part of the treatment process (Ellis & Dryden, 2007).

It can be said that REBT is an approach anchored in the present, but not to the same degree as mindfulness. REBT-based homework, for example, often asks patients to reflect on how they were disturbed in the recent past, and to recall the thoughts, emotions, and behavioural tendencies they experienced during that disturbance. Moreover, Ellis considered mindfulness’ emphasis on focusing only on the present to be unnecessarily extreme: “Why shouldn’t you at times want to get somewhere else?” he wrote (Ellis, 2006, p. 68). Ellis openly revelled in future-oriented thought endeavours and suggested that that staying in the moment may only be adaptive for stressed individuals who were constantly worrying about the future. He even expressed concern that a heavy emphasis on the present could lead some individuals to develop a compulsive habit (Ellis, 2006).

Dredze (2020) suggested that rather than clashing with REBT techniques, “these [present-focussed mindfulness] interventions may actually be firmly rooted in core REBT methodology” (p. 324). Mindfulness’ emphasis on the present may support functional disputation, he posited, a key REBT technique, and therefore this could bridge between the seemingly divergent positions of REBT and mindfulness. Functional disputes in REBT are based on the utility or benefits of maintaining a certain belief (Neenan & Dryden, 1999). Mindfulness philosophy may thus align with a functional disputation undertaken by a client that is examining how dwelling on past mistakes and failures will help them to achieve their current goals and aspirations, Dredze (2020) suggested. Dwelling unnecessarily on the past would be considered irrational within an REBT framework, as would demandingness relating to future-based outcomes. Perhaps mindfulness techniques could be additive alongside REBT in anchoring an individual to the present, whereupon they can still undertake adaptive, realistic, and goal-oriented planning and action. For example, by paying attention to their present experience, mindfulness meditation could help an athlete become less attached to past competition failures, allowing them to recognise that the only thing which truly exists is the here and now. From which, they could examine their current irrational beliefs about failure more broadly, better equipping them for inevitable future failures.

An interesting line of research explored in recent years as brain scanning capabilities have enhanced has been mindfulness meditation’s potential to decrease unhelpful mind wandering by deactivating the Default-Mode Network (DMN; Brewer et al., 2011). Mind wandering is extremely common but has been associated with lower levels of happiness, and higher DMN activity (Brewer et al., 2011; Killingsworth & Gilbert, 2010). It seems reasonable to consider that individuals who are more ‘anchored’ in awareness of the present moment, with less mind wandering, may have greater capacity to undertake effective irrational belief identification, disputation, and achieve rational emotional insight with REBT. If true, this is another reason why mindfulness meditation might be a useful additional tool in the REBT practitioner toolbox.

### Meditation

REBT and mindfulness take different approaches to meditation. In mindfulness-based therapeutic approaches, such as MBSR and MBCT, meditation is used as the primary means of practicing non-judgemental present moment awareness. Intellectual understanding of the principles of mindfulness is considered insufficient to enact emotional and philosophic change; rather, meditation is seen as necessary to bring about such change. During meditation, participants are encouraged to recognise distracting or ruminative thoughts and to let them go by coming back to physical anchor points such as the breath, feet, seat, and hands (Segal et al., 2002). Most third wave CBT interventions include meditation, but some emphasise it more than others. With MBCT and MBSR, for example, meditation is the central component of training (Segal et al., 2002). Meditation is included within ACT and DBT, but each also includes non-meditative aspects (Hayes et al., 1999; Linehan, 1993). ACT, for example, employs a range of mindfulness-based experiential exercises which are non-meditative to instil its central teachings on acceptance, cognitive diffusion, present moment awareness and self as context (Hayes et al., 1999).

REBT does not include meditation, but nor is it precluded: “With REBT, you may or may not meditate”, noted Ellis (2006 p. 64). While Buddhism (forbearer of contemporary mindfulness) and Stoicism (influential in REBT) both traditionally employed meditation, the purpose of meditation was different for each. The philosopher Nancy Sherman (2005) noted that a Stoic’s conceptualisation of meditation was one of inward rational enquiry, where the enquiring Stoic would actively review their behaviour and beliefs at the end of the day. A Zen Buddhist undertaking mindfulness meditation, on the other hand, would seek to dispense with argument and rational thought and reach instead a higher plane of awareness, transcendent of active judgement (Sherman, 2005). Meditative practice, albeit with a differing focus, has thus long been integral to both ancient Stoic and Buddhist practice though it is more typically associated with the latter than the former.

## REBT and Mindfulness in High Performance Settings

High performance settings, such as sport, business, the military, healthcare, and the arts require individuals to execute complex and demanding tasks skilfully and consistently under pressure. To be successful in such settings, individuals need not only to develop a refined set of practical, often physical ‘tools’, but also psychological and emotional regulation capacities. To illustrate, Jones and colleagues’ (2009) High Performance Environment (HPE) Model identifies the psychological and social factors which lead to sustainable high performance at individual, group, and organisational levels. In addition to areas of leadership and organisational climate, Jones and colleagues identify individual capacity as being a key driver of performance, specifically emotional intelligence, described as “the ability to perceive, understand and regulate emotions” and mental toughness, “the ability to perform to high standards through times of personal and professional pressure” (Jones et al., 2009, p. 144).

As outlined, REBT and mindfulness appear to bring overlapping qualities and perspectives to enhancing emotional regulation, and thus future researchers might investigate how they could work together in high performance settings to improve outcomes. To inform future researchers, we will consider how REBT and mindfulness might integrate in sport and business, two high performance settings where both approaches have already been applied separately but not together. These ideas are offered to stimulate future discussion and thinking as relates to integrating REBT and mindfulness in high performance settings. It is not our intention to provide a complete framework, to attempt to do so would be beyond the scope of this commentary.

### REBT and Mindfulness in Sport

REBT in sporting contexts focuses on helping athletes to examine and dispute their irrational beliefs, ultimately replacing them with rational alternatives to aid their goal progression and emotional wellbeing (Turner, 2019). According to REBT theory, unhealthy emotional disturbances inhibit athlete goal achievement (as well as general wellbeing), and result from irrational beliefs relating to some or all of *demandingness* (i.e., primary ‘musts’, such as ‘I must win’), *awfulizing* (e.g., ‘it would be awful if I do not win’), *low frustration tolerance* (e.g., ‘I cannot cope if I do not win’) and *global rating/damning* (e.g., ‘If I don’t win, it means I am a failure as a person’; Turner, 2019). It is not difficult to see how holding such irrational beliefs—underpinned by rigidity—could lead to performance-inhibiting unhealthy negative emotions; for example, anxiety in the build-up to or during a sporting performance, or depression afterwards if demanded outcomes are not met in competition.

There may be several ways in which mindfulness practice could work alongside REBT in high performance sport to enhance psychological and emotional regulation capacity and contribute to improved performance outcomes. These are revealed within the overlaps between the two approaches as identified previously in this commentary, particularly awareness and acceptance. For example, mindfulness-based practice may help athletes by enhancing their metacognitive capacity; an ability to step back and observe, or be aware of, internal thought processes clearly without getting caught up or distracted by them (Torneatto, 2002). This could help athletes to experientially ‘witness’ the causal relationship between their irrational beliefs at ‘B’ and their emotional outcomes at ‘C’, abetting the irrational belief disputation process and pursuit of rational emotional insight within an REBT framework. Additionally, both in training and the heat of sporting competition, mindfulness could enhance an athlete’s capacity to allow unpleasant and potentially distracting thoughts to arise and dissipate without becoming involved in or overwhelmed by a potentially draining and distracting mental ‘battle’ with them (at ‘C’ in the ABC model of REBT). This capacity to mindfully observe and accept the presence of thoughts and feelings as they arise and dissipate at ‘C’ could dovetail with irrational belief disputation work (at ‘B’) undertaken by athletes with coaches/psychologists at appropriate times, perhaps in between competition.

One of the central philosophical teachings of mindfulness, namely the idea of impermanence, may also be integrated with REBT for athletes. Mindfulness philosophy teaches that *everything is always changing*, both in terms of the world around us, but also the thoughts and feelings we experience internally (Lee, 2018). Mindfulness instils this lesson through meditation, where the practitioner observes thoughts and feelings as they come and go in the present moment (Lee, 2018). An athlete who has internalised the teaching of impermanence may theoretically be more at ease in accepting their thoughts and feelings at ‘C’ as they arise in preparation for and during competition and thus able to remain focused on holding rational beliefs at ‘B’ in pursuit of goals. The concept of impermanence may also enrich an athlete’s overarching rational emotional insight with regards to USA, UOA and ULA within the REBT framework, helping them to cope with (by accepting) the emotional ups and downs that are an inevitable part of an athlete’s life.

In complex high-pressure environments such as sport, staying grounded and task-focussed is key to optimum performance as well as self-care. There is an array of practical mindfulness exercises which athletes may employ in preparation for and during competition, and which could work effectively *alongside* cognitive restructuring work with REBT. For example, a regular mindfulness meditation practice may provide the athlete with an enhanced overall capacity to anchor one’s attention to the present, and to reduce mind wandering. In particularly high demand situations, an athlete may use a mindfulness 3-step (also known as three minute) breathing space (Segal et al., 2018) technique to orient themselves to the present moment, reduce distraction from unhelpful thoughts, and enable more skilful responses. In addition, or alternatively, athletes could be encouraged to bring mindful awareness to their senses, for example by noticing things that they can smell, hear, see, taste, and feel, during moments when they feel distracted by ruminative thoughts or unfocused during a performance situation. Mindfulness practices of this nature may enhance an athlete’s likelihood of achieving flow, a sought-after state wherein athletes become entirely ‘present’ in the delivery of their performance, absent of past-or-future-related thinking, and which has been linked to enhanced sporting performance outcomes (e.g. Aherne et al., 2011).

### REBT and Mindfulness in Business

Millions of people worldwide work in highly pressurised and complex business environments where high productively and goal achievement is necessary for success. Workplace stress and employee mental ill health within such settings is a known problem, and its human and economic cost is substantial and well documented (Chisholm et al., 2016). The literature is clear that mentally healthy employees perform better at work, which leads in turn to enhanced individual and overall business outcomes (e.g., Krekel et al., 2019). Finding ways to enhance mental wellbeing in business and workplace settings is therefore of great importance.

Many, if not all, of the previously suggested areas where mindfulness may support REBT in sporting settings could also be applied to business. As with athletes, it has been observed that irrational beliefs have a detrimental effect on the performance and wellbeing of individuals working within business settings. Unhealthy emotions such as anxiety, anger, shame, envy, and guilt are prevalent in business settings and contribute towards chronic stress and poorer performance outcomes (Bernard, 2019; Criddle, 2007; Turner & Barker, 2015). In the same way as explained within the context of sport, mindfulness may help enhance REBT-based outcomes with business executives by strengthening a metacognitive capacity to observe ‘B to C’ connections experientially.

Similarly, an executive being taught to accept themselves, others, and life unconditionally through REBT may also benefit from a mindfulness practice that enhances their capacity to observe and accept thoughts and emotions non-judgementally as they arise and dissipate, in the knowledge that they are impermanent and not ‘facts’ but merely mental and bodily events. Such a philosophy may enhance an executive’s capacity to, for example, engage constructively with a manager despite transient feelings and thoughts related to the unhealthy negative emotion of anger, take on a new project or deliver (rather than avoid) a presentation despite the presence of anxious thoughts and feelings, or make high quality rational strategic decisions in the best interests of the business despite the presence of unhealthy guilt.

In the same way as with athletes, mindfulness techniques could potentially dovetail with REBT interventions in business settings to help individuals stay grounded and task-focused during high pressure moments. For example, it is common for executives to experience anxiety in relation to public speaking at work (Linden & Muschalla, 2007). An executive in such a situation would be helped within an REBT framework to identify, dispute, and replace his or her irrational ‘primary must’ belief (which could be: “my colleagues must think that I am capable and in control at all times”) and any accompanying awfulizing, low frustration tolerance, and self-damning-related beliefs (Criddle, 2007). Mindfulness practices could potentially add to the belief change work undertaken with REBT by equipping the individual to remain undistracted or perturbed by unhelpful ruminative thinking and anchored to the present during the run up to and the course of presentation delivery. Mindfulness techniques such as the 3-step breathing space, noticing senses, or body scanning—all of which ground the individual to the present and reduce unhelpful mind wandering—may be helpful techniques for an executive to employ in advance of (or during, as appropriate) this high-performance, high pressure situation.

Mindfulness was identified by Toniolo-Barrios &Pitt (2021) as a potentially beneficial practice for employees to engage in as a way of coping with the complexity, uncertainty, and stress of working remotely through the Covid-19 pandemic. They noted that mindfulness practices such as breathing spaces, body scanning, meditation and mindful ‘check-ins’ could help executives to manage ‘Zoom fatigue’, enhance focus, and switch off from work at the end of the day (Toniolo-Barrios & Pitt, 2021). These seem like eminently sensible and viable suggestions, ones which apply just as readily to the complexities of post-pandemic working, and which could be added to or integrated with belief change work within an REBT framework as and when necessary. This example, and the others outlined above, serve to highlight the potential for mindfulness and REBT techniques to be used together to enhance the performance and mental wellbeing of executives in business settings and athletes in preparation for and during competition.

## Conclusions and Future Directions

To conclude, REBT and mindfulness-based therapeutic interventions have followed similar trajectories having been introduced to help with clinical disorders originally, before later being considered within non-clinical and high performance settings. To date, there is scant evidence of integrated REBT and mindfulness approaches in clinical, non-clinical or high-performance settings, despite apparent overlaps in their philosophical approaches and despite aspects of cognitive therapy and mindfulness having become integrated within third wave CBT approaches. Overlaps between REBT and mindfulness explored in this commentary relate to the causes of disturbance, themes of acceptance, and enhancing awareness and functioning in line with reality. Divergences explored relate to their respective positions on judgement of thoughts, the present moment, and meditation. These diveregences are not considered barriers to integration, but should be considered by practitioners seeking to use REBT and mindfulness in combination.

The literature suggests REBT is generally effective as a standalone intervention in clinical settings and in high performance settings, although it is more convincing in the in the former than the latter by virtue of its richer heritage. It could be reasonably argued that REBT functions sufficiently without needing to incorporate mindfulness. However REBT, like all psychological approaches, is imperfect, and there appear to be sound reasons to consider that mindfulness might help to enhance outcomes from REBT in the context of high performance (and potentially beyond) if integrated skilfully.

In this commentary, the high-performance settings of sport and business are offered as examples of where REBT and mindfulness could be integrated to aid both psychological performance and wellbeing. It is considered that mindfulness practice may have potential to deliver an enhanced experiential viewpoint into how beliefs influence cognitive, emotional, and behavioural outcomes, which could ultimately support an individual’s transition from cognitive to emotional rational insight, something which REBT practitioners recognise as being notoriously difficult to do (Neenan & Dryden, 2004).

A hypothetically ‘mindful rational’ athlete or executive could be one who has fostered an allowing and more harmonious relationship with unwanted thoughts and feelings (at ‘C’ in REBT’s ABC model), helping them to remain present, aware and focused in the heat of sporting or business ‘battle’. By simultaneously holding rational beliefs about themselves, others, and life (at ‘B’)—acquired through timely REBT-based enquiry—the ‘mindful rational’ athlete or executive would also be well placed to respond flexibly and adaptively in the presence of adversity, aiding the pursuit of goals. Future research may shed light on whether such integrated qualities could contribute to enhanced performance—and emotional health—in either setting.

## Data Availability

Not applicable.
